# Tetra­pyridine­bis(trichloro­acetato)nickel(II)

**DOI:** 10.1107/S1600536809030025

**Published:** 2009-08-08

**Authors:** Li-Min Li, Fang-Fang Jian, Xiao-Yan Ren

**Affiliations:** a Microscale Science Institute, Department of Chemistry and Chemical Engineering, Weifang University, Weifang 261061, People’s Republic of China; b Microscale Science Institute, Weifang University, Weifang 261061, People’s Republic of China

## Abstract

The title compound, [Ni(C_2_Cl_3_O_2_)_2_(C_5_H_5_N)_4_], was prepared by the reaction of pyridine and trichloro­acetatonickel(II) in ethanol solution at room temperature. The Ni^II^ atom is located on a twofold rotation axis and has a slightly distorted octa­hedral coordination made up of four N atoms of the pyridine ligands and two O atoms of trichloro­acetate anions. The mol­ecular structure and packing are stabilized by intra- and inter­molecular C—H⋯O hydrogen-bonding inter­actions.

## Related literature

For the structural and magnetic properties of transition metal complexes involving a pyridine or a substituted pyridine ligand, see: Crawford & Hatfield (1977 ▶); Marsh *et al.* (1981 ▶); Swank & Willett (1980 ▶). For Ni—O and Ni—N bond lengths, see: Bentiss *et al.* (2002 ▶); Rodopoulos *et al.* (2001 ▶).
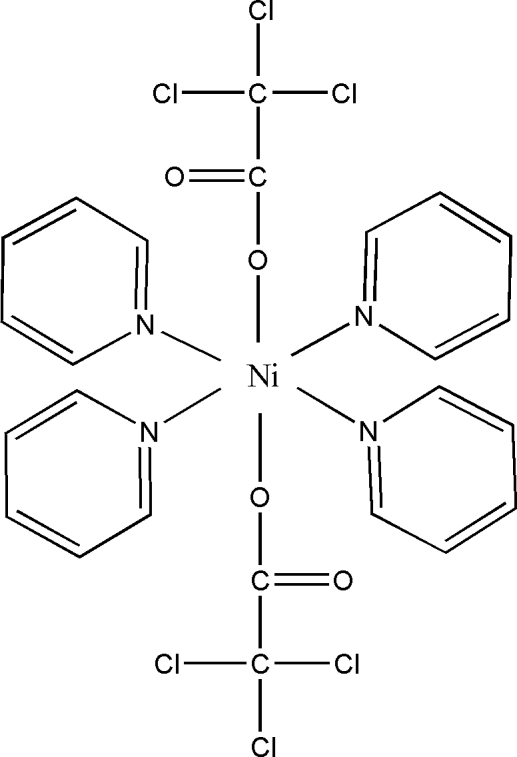


## Experimental

### 

#### Crystal data

[Ni(C_2_Cl_3_O_2_)_2_(C_5_H_5_N)_4_]
*M* *_r_* = 699.85
Monoclinic, 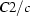

*a* = 9.1073 (18) Å
*b* = 17.078 (3) Å
*c* = 19.376 (6) Å
β = 106.94 (3)°
*V* = 2882.9 (12) Å^3^
*Z* = 4
Mo *K*α radiation
μ = 1.27 mm^−1^
*T* = 293 K
0.30 × 0.20 × 0.10 mm

#### Data collection

Bruker SMART CCD area-detector diffractometer
Absorption correction: none
13819 measured reflections
3312 independent reflections
2898 reflections with *I* > 2σ(*I*)
*R*_int_ = 0.054

#### Refinement

*R*[*F* ^2^ > 2σ(*F* ^2^)] = 0.064
*wR*(*F* ^2^) = 0.187
*S* = 1.07
3312 reflections
177 parameters
H-atom parameters constrained
Δρ_max_ = 1.63 e Å^−3^
Δρ_min_ = −1.04 e Å^−3^

### 

Data collection: *SMART* (Bruker, 1997 ▶); cell refinement: *SAINT* (Bruker, 1997 ▶); data reduction: *SAINT*; program(s) used to solve structure: *SHELXS97* (Sheldrick, 2008 ▶); program(s) used to refine structure: *SHELXL97* (Sheldrick, 2008 ▶); molecular graphics: *SHELXTL* (Sheldrick, 2008 ▶); software used to prepare material for publication: *SHELXTL*.

## Supplementary Material

Crystal structure: contains datablocks global, I. DOI: 10.1107/S1600536809030025/at2853sup1.cif

Structure factors: contains datablocks I. DOI: 10.1107/S1600536809030025/at2853Isup2.hkl

Additional supplementary materials:  crystallographic information; 3D view; checkCIF report

## Figures and Tables

**Table 1:** Hydrogen-bond geometry (Å, °)

*D*—H⋯*A*	*D*—H	H⋯*A*	*D*⋯*A*	*D*—H⋯*A*
C4*A*—H4*AA*⋯O1^i^	0.93	2.55	3.442 (7)	162
C1*B*—H1*BA*⋯O2	0.93	2.59	2.943 (6)	103
C1*A*—H1*AA*⋯O1^ii^	0.93	2.41	3.253 (7)	151

Symmetry codes: (i) 
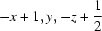
; (ii) 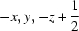.

**Fig. 1.**
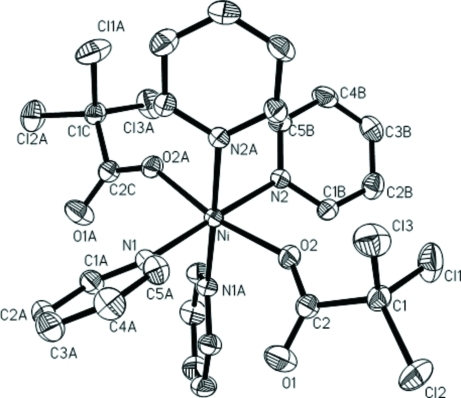
The structure of the title compound showing 30% probability displacement ellipsoids and the atom-numbering scheme.

## supplementary crystallographic information

### Comment

The structural and magnetic properties of transition metal complexes in the solid state have been the subjects of numerous recent publications. This is particularly true for the cases where *L* is pyridine or a substituted pyridine (Swank & Willett, 1980; Marsh *et al.*, 1981; Crawford & Hatfield, 1977). Much of this work has been concerned with the correlation of the structural properties of these complexes with their magnetic properties. In order to search for new complexes of this type, we synthesized the title compound and report its crystal structure here.

The title compound contains one nickel(II), four pyridine ligands and two trichloroacetic acid molecules. The coordination sphere of the nickel(II) ion is best described as a slightly distorted octahedron. The Ni—O and Ni—N bond lengths are in agreement with those reported recently (Bentiss *et al.*, 2002; Rodopoulos *et al.*, 2001). The crystal packing is stabilized by C—H···O intra- and intermolecular hydrogen interaction (Table 1).

### Experimental

The title compound was obtained by adding pyridine (4 mmol) dropwise to a solution of nickel(II) trichloroacetic acid (1 mmol) in ethanol (30 ml) under stirred for 1 h at room temperature. A green solution was formed and after a few days block crystals precipitated.

### Refinement

H atoms were fixed geometrically and allowed to ride on their attached atoms, with C—H and N—H distances of 0.93–0.96 and 0.86 Å, and with *U*_iso_ = 1.2*U*_eq_.

### Crystal data

**Table:**

[Ni(C_2_Cl_3_O_2_)_2_(C_5_H_5_N)_4_]	*F*(000) = 1416
*M**_r_* = 699.85	*D*_x_ = 1.612 Mg m^−^^3^
Monoclinic, *C*2/*c*	Mo *K*α radiation, λ = 0.71073 Å
Hall symbol: -C 2yc	Cell parameters from 2898 reflections
*a* = 9.1073 (18) Å	θ = 3.1–27.5°
*b* = 17.078 (3) Å	µ = 1.27 mm^−^^1^
*c* = 19.376 (6) Å	*T* = 293 K
β = 106.94 (3)°	Block, green
*V* = 2882.9 (12) Å^3^	0.30 × 0.20 × 0.10 mm
*Z* = 4	

### Data collection

**Table:**

Bruker SMART CCD area-detector diffractometer	2898 reflections with *I* > 2σ(*I*)
Radiation source: fine-focus sealed tube	*R*_int_ = 0.054
graphite	θ_max_ = 27.5°, θ_min_ = 3.1°
φ and ω scans	*h* = −11→11
13819 measured reflections	*k* = −22→22
3312 independent reflections	*l* = −25→25

### Refinement

**Table:**

Refinement on *F*^2^	Primary atom site location: structure-invariant direct methods
Least-squares matrix: full	Secondary atom site location: difference Fourier map
*R*[*F*^2^ > 2σ(*F*^2^)] = 0.064	Hydrogen site location: inferred from neighbouring sites
*wR*(*F*^2^) = 0.187	H-atom parameters constrained
*S* = 1.07	*w* = 1/[σ^2^(*F*_o_^2^) + (0.0862*P*)^2^ + 12.4612*P*] where *P* = (*F*_o_^2^ + 2*F*_c_^2^)/3
3312 reflections	(Δ/σ)_max_ < 0.001
177 parameters	Δρ_max_ = 1.63 e Å^−^^3^
0 restraints	Δρ_min_ = −1.04 e Å^−^^3^

### Special details

**Table:**

Geometry. All e.s.d.'s (except the e.s.d. in the dihedral angle between two l.s. planes) are estimated using the full covariance matrix. The cell e.s.d.'s are taken into account individually in the estimation of e.s.d.'s in distances, angles and torsion angles; correlations between e.s.d.'s in cell parameters are only used when they are defined by crystal symmetry. An approximate (isotropic) treatment of cell e.s.d.'s is used for estimating e.s.d.'s involving l.s. planes.
Refinement. Refinement of *F*^2^ against ALL reflections. The weighted *R*-factor *wR* and goodness of fit *S* are based on *F*^2^, conventional *R*-factors *R* are based on *F*, with *F* set to zero for negative *F*^2^. The threshold expression of *F*^2^ > σ(*F*^2^) is used only for calculating *R*-factors(gt) *etc*. and is not relevant to the choice of reflections for refinement. *R*-factors based on *F*^2^ are statistically about twice as large as those based on *F*, and *R*- factors based on ALL data will be even larger.

### Fractional atomic coordinates and isotropic or equivalent isotropic displacement parameters (Å^2^)

**Table:**

	*x*	*y*	*z*	*U*_iso_*/*U*_eq_	
Ni	0.0000	0.03441 (4)	0.2500	0.0324 (2)	
Cl1	0.1137 (2)	0.13260 (11)	0.03902 (9)	0.0835 (5)	
Cl2	0.3294 (2)	0.01282 (11)	0.02840 (8)	0.0816 (5)	
Cl3	0.40222 (17)	0.11568 (9)	0.14971 (9)	0.0755 (4)	
O1	0.2031 (5)	−0.0615 (2)	0.1326 (2)	0.0663 (10)	
O2	0.1059 (4)	0.04555 (17)	0.16902 (15)	0.0444 (7)	
N2	−0.1503 (4)	0.12325 (18)	0.19207 (16)	0.0366 (7)	
N1	0.1589 (4)	−0.05134 (18)	0.30435 (17)	0.0369 (7)	
C1	0.2501 (5)	0.0635 (3)	0.0878 (2)	0.0467 (9)	
C3B	−0.3058 (6)	0.2490 (3)	0.1124 (3)	0.0587 (12)	
H3BA	−0.3558	0.2915	0.0857	0.070*	
C2A	0.2185 (6)	−0.1699 (3)	0.3717 (3)	0.0556 (11)	
H2AA	0.1850	−0.2112	0.3946	0.067*	
C3A	0.3683 (6)	−0.1660 (3)	0.3713 (3)	0.0603 (12)	
H3AA	0.4381	−0.2046	0.3936	0.072*	
C2	0.1764 (4)	0.0082 (3)	0.1343 (2)	0.0406 (8)	
C4B	−0.2629 (6)	0.2506 (3)	0.1859 (3)	0.0565 (12)	
H4BA	−0.2852	0.2940	0.2101	0.068*	
C4A	0.4137 (5)	−0.1035 (3)	0.3372 (3)	0.0575 (12)	
H4AA	0.5147	−0.0992	0.3360	0.069*	
C2B	−0.2737 (6)	0.1829 (3)	0.0785 (3)	0.0565 (11)	
H2BA	−0.3042	0.1796	0.0284	0.068*	
C5B	−0.1861 (5)	0.1875 (3)	0.2240 (2)	0.0458 (9)	
H5BA	−0.1577	0.1894	0.2741	0.055*	
C5A	0.3063 (5)	−0.0477 (3)	0.3048 (3)	0.0482 (10)	
H5AA	0.3376	−0.0055	0.2823	0.058*	
C1B	−0.1961 (5)	0.1222 (3)	0.1197 (2)	0.0445 (9)	
H1BA	−0.1742	0.0781	0.0963	0.053*	
C1A	0.1178 (5)	−0.1121 (2)	0.3380 (2)	0.0452 (9)	
H1AA	0.0162	−0.1155	0.3386	0.054*	

### Atomic displacement parameters (Å^2^)

**Table:**

	*U*^11^	*U*^22^	*U*^33^	*U*^12^	*U*^13^	*U*^23^
Ni	0.0382 (4)	0.0279 (3)	0.0332 (3)	0.000	0.0136 (3)	0.000
Cl1	0.0853 (10)	0.0995 (12)	0.0730 (9)	0.0308 (9)	0.0347 (8)	0.0410 (8)
Cl2	0.0984 (11)	0.0965 (11)	0.0680 (8)	0.0099 (9)	0.0529 (8)	−0.0121 (8)
Cl3	0.0637 (8)	0.0730 (9)	0.0956 (11)	−0.0224 (7)	0.0323 (7)	−0.0145 (8)
O1	0.070 (2)	0.0427 (17)	0.100 (3)	−0.0004 (16)	0.047 (2)	−0.0027 (18)
O2	0.0524 (16)	0.0448 (16)	0.0427 (15)	0.0042 (13)	0.0245 (13)	0.0029 (12)
N2	0.0428 (17)	0.0319 (15)	0.0361 (15)	0.0023 (13)	0.0130 (13)	0.0004 (12)
N1	0.0396 (16)	0.0324 (15)	0.0386 (16)	0.0006 (12)	0.0114 (13)	0.0020 (12)
C1	0.051 (2)	0.050 (2)	0.045 (2)	0.0052 (19)	0.0234 (18)	0.0020 (18)
C3B	0.060 (3)	0.046 (2)	0.067 (3)	0.009 (2)	0.014 (2)	0.015 (2)
C2A	0.067 (3)	0.045 (2)	0.057 (3)	0.009 (2)	0.020 (2)	0.011 (2)
C3A	0.059 (3)	0.057 (3)	0.059 (3)	0.018 (2)	0.008 (2)	0.006 (2)
C2	0.0380 (19)	0.044 (2)	0.042 (2)	−0.0003 (16)	0.0146 (16)	0.0001 (16)
C4B	0.066 (3)	0.036 (2)	0.068 (3)	0.011 (2)	0.020 (2)	−0.002 (2)
C4A	0.038 (2)	0.064 (3)	0.067 (3)	0.003 (2)	0.010 (2)	0.003 (2)
C2B	0.057 (3)	0.066 (3)	0.044 (2)	0.007 (2)	0.0107 (19)	0.009 (2)
C5B	0.051 (2)	0.041 (2)	0.047 (2)	0.0073 (18)	0.0161 (18)	−0.0020 (17)
C5A	0.040 (2)	0.049 (2)	0.055 (2)	−0.0046 (17)	0.0125 (18)	0.0059 (19)
C1B	0.053 (2)	0.043 (2)	0.0371 (19)	0.0042 (18)	0.0130 (17)	−0.0007 (16)
C1A	0.047 (2)	0.040 (2)	0.052 (2)	0.0021 (17)	0.0204 (18)	0.0031 (18)

### Geometric parameters (Å, °)

**Table:**

Ni—O2	2.076 (3)	C3B—C2B	1.379 (7)
Ni—O2^i^	2.076 (3)	C3B—H3BA	0.9300
Ni—N1^i^	2.112 (3)	C2A—C3A	1.368 (8)
Ni—N1	2.112 (3)	C2A—C1A	1.377 (6)
Ni—N2^i^	2.131 (3)	C2A—H2AA	0.9300
Ni—N2	2.131 (3)	C3A—C4A	1.381 (8)
Cl1—C1	1.773 (5)	C3A—H3AA	0.9300
Cl2—C1	1.754 (4)	C4B—C5B	1.377 (6)
Cl3—C1	1.784 (5)	C4B—H4BA	0.9300
O1—C2	1.217 (5)	C4A—C5A	1.378 (7)
O2—C2	1.234 (5)	C4A—H4AA	0.9300
N2—C1B	1.341 (5)	C2B—C1B	1.373 (6)
N2—C5B	1.346 (5)	C2B—H2BA	0.9300
N1—C1A	1.335 (5)	C5B—H5BA	0.9300
N1—C5A	1.342 (5)	C5A—H5AA	0.9300
C1—C2	1.585 (6)	C1B—H1BA	0.9300
C3B—C4B	1.362 (8)	C1A—H1AA	0.9300
			
O2—Ni—O2^i^	169.48 (17)	C3A—C2A—C1A	119.3 (5)
O2—Ni—N1^i^	95.06 (13)	C3A—C2A—H2AA	120.3
O2^i^—Ni—N1^i^	92.23 (12)	C1A—C2A—H2AA	120.3
O2—Ni—N1	92.23 (12)	C2A—C3A—C4A	118.6 (4)
O2^i^—Ni—N1	95.06 (13)	C2A—C3A—H3AA	120.7
N1^i^—Ni—N1	92.18 (18)	C4A—C3A—H3AA	120.7
O2—Ni—N2^i^	87.95 (12)	O1—C2—O2	131.3 (4)
O2^i^—Ni—N2^i^	84.56 (12)	O1—C2—C1	116.6 (4)
N1^i^—Ni—N2^i^	176.54 (12)	O2—C2—C1	112.1 (4)
N1—Ni—N2^i^	89.39 (13)	C3B—C4B—C5B	119.4 (4)
O2—Ni—N2	84.56 (12)	C3B—C4B—H4BA	120.3
O2^i^—Ni—N2	87.95 (12)	C5B—C4B—H4BA	120.3
N1^i^—Ni—N2	89.39 (13)	C5A—C4A—C3A	118.7 (4)
N1—Ni—N2	176.54 (12)	C5A—C4A—H4AA	120.6
N2^i^—Ni—N2	89.20 (17)	C3A—C4A—H4AA	120.6
C2—O2—Ni	142.6 (3)	C1B—C2B—C3B	119.0 (4)
C1B—N2—C5B	116.7 (3)	C1B—C2B—H2BA	120.5
C1B—N2—Ni	119.8 (3)	C3B—C2B—H2BA	120.5
C5B—N2—Ni	122.9 (3)	N2—C5B—C4B	123.0 (4)
C1A—N1—C5A	117.1 (4)	N2—C5B—H5BA	118.5
C1A—N1—Ni	122.2 (3)	C4B—C5B—H5BA	118.5
C5A—N1—Ni	120.7 (3)	N1—C5A—C4A	123.2 (4)
C2—C1—Cl2	113.7 (3)	N1—C5A—H5AA	118.4
C2—C1—Cl1	110.6 (3)	C4A—C5A—H5AA	118.4
Cl2—C1—Cl1	109.7 (2)	N2—C1B—C2B	123.3 (4)
C2—C1—Cl3	106.8 (3)	N2—C1B—H1BA	118.4
Cl2—C1—Cl3	107.5 (2)	C2B—C1B—H1BA	118.4
Cl1—C1—Cl3	108.2 (3)	N1—C1A—C2A	123.2 (4)
C4B—C3B—C2B	118.6 (4)	N1—C1A—H1AA	118.4
C4B—C3B—H3BA	120.7	C2A—C1A—H1AA	118.4
C2B—C3B—H3BA	120.7		
			
O2^i^—Ni—O2—C2	167.2 (5)	Ni—O2—C2—C1	−169.6 (3)
N1^i^—Ni—O2—C2	−59.1 (5)	Cl2—C1—C2—O1	11.1 (5)
N1—Ni—O2—C2	33.3 (5)	Cl1—C1—C2—O1	135.1 (4)
N2^i^—Ni—O2—C2	122.6 (5)	Cl3—C1—C2—O1	−107.3 (4)
N2—Ni—O2—C2	−148.0 (5)	Cl2—C1—C2—O2	−171.9 (3)
O2—Ni—N2—C1B	39.1 (3)	Cl1—C1—C2—O2	−47.9 (4)
O2^i^—Ni—N2—C1B	−148.3 (3)	Cl3—C1—C2—O2	69.7 (4)
N1^i^—Ni—N2—C1B	−56.0 (3)	C2B—C3B—C4B—C5B	1.4 (8)
N2^i^—Ni—N2—C1B	127.1 (3)	C2A—C3A—C4A—C5A	0.0 (8)
O2—Ni—N2—C5B	−131.3 (3)	C4B—C3B—C2B—C1B	−1.8 (8)
O2^i^—Ni—N2—C5B	41.3 (3)	C1B—N2—C5B—C4B	−1.3 (6)
N1^i^—Ni—N2—C5B	133.5 (3)	Ni—N2—C5B—C4B	169.4 (4)
N2^i^—Ni—N2—C5B	−43.3 (3)	C3B—C4B—C5B—N2	0.2 (8)
O2—Ni—N1—C1A	−143.2 (3)	C1A—N1—C5A—C4A	0.9 (7)
O2^i^—Ni—N1—C1A	44.4 (3)	Ni—N1—C5A—C4A	−177.6 (4)
N1^i^—Ni—N1—C1A	−48.1 (3)	C3A—C4A—C5A—N1	−0.6 (8)
N2^i^—Ni—N1—C1A	128.8 (3)	C5B—N2—C1B—C2B	0.9 (6)
O2—Ni—N1—C5A	35.1 (3)	Ni—N2—C1B—C2B	−170.1 (4)
O2^i^—Ni—N1—C5A	−137.3 (3)	C3B—C2B—C1B—N2	0.6 (8)
N1^i^—Ni—N1—C5A	130.3 (4)	C5A—N1—C1A—C2A	−0.5 (6)
N2^i^—Ni—N1—C5A	−52.8 (3)	Ni—N1—C1A—C2A	177.9 (4)
C1A—C2A—C3A—C4A	0.3 (8)	C3A—C2A—C1A—N1	−0.1 (7)
Ni—O2—C2—O1	6.8 (8)		

Symmetry codes: (i) −*x*, *y*, −*z*+1/2.

### Hydrogen-bond geometry (Å, °)

**Table:**

*D*—H···*A*	*D*—H	H···*A*	*D*···*A*	*D*—H···*A*
C4A—H4AA···O1^ii^	0.93	2.55	3.442 (7)	162
C1B—H1BA···O2	0.93	2.59	2.943 (6)	103
C1A—H1AA···O1^iii^	0.93	2.41	3.253 (7)	151

Symmetry codes: (ii) −*x*+1, *y*, −*z*+1/2; (iii) −*x*, *y*, −*z*+1/2.
